# Initial Adjustment to the COVID-19 Pandemic and the Associated Shutdown in Children and Adolescents With Chronic Pain and Their Families

**DOI:** 10.3389/fpain.2021.713430

**Published:** 2021-09-30

**Authors:** Karen J. Kaczynski, Cindy Yu Hsing Chang, Justin Chimoff, Camila Koike, Charles B. Berde, Deirdre E. Logan, Sarah Nelson, Joe Kossowsky

**Affiliations:** ^1^Department of Anesthesiology, Critical Care and Pain Medicine, Harvard Medical School, Boston Children's Hospital, Boston, MA, United States; ^2^Department of Psychiatry, Harvard Medical School, Boston, MA, United States

**Keywords:** pediatric pain, pain catastrophizing, COVID-19, anxiety, functional disability

## Abstract

**Objectives:** Youth with chronic pain often struggle to function in multiple domains due to pain and associated psychosocial distress. In 2020, schools and businesses shut down and people were encouraged to remain at home due to the COVID-19 pandemic, eliminating or reducing stress due to functional difficulties. This study assessed whether pain and associated psychosocial outcomes improved in youth with chronic pain during the shutdown, compared with before the pandemic.

**Methods:** Patients who completed clinical outcome measures during a multidisciplinary evaluation before the pandemic were readministered the same measures (PROMIS Anxiety, Depression, Sleep Disturbance, PCS, PedsQL) during the shutdown. At follow-up, patients also completed measures of adjustment to COVID-19 and their parents completed a measure of pandemic effects.

**Results:** Participants included 47 patients ages 8–18 and a parent/guardian. The pandemic impacted families in both positive (e.g., more quality time with family) and negative ways (e.g., social isolation, disruption in care). Pain intensity and pain catastrophizing significantly decreased during the shutdown (*ps* <0.01). Change in pain catastrophizing was correlated positively with change in psychological stress (*p* = 0.004) and anxiety (*p* = 0.005) and negatively with change in quality of life (*p* = 0.024).

**Discussion:** Pain and pain catastrophizing decreased initially during the shutdown related to the COVID-19 pandemic. Change in catastrophizing was associated with change in stress and anxiety. It may be that the reduction in functional demands contributed to this change. Functional difficulties should be addressed in treatment, including pain coping and also environmental modification to support optimal functioning in youth with chronic pain.

## Introduction

Chronic pain is common in pediatrics, affecting up to 37.3% of children and adolescent (1). The biopsychosocial model is helpful for understanding the complex interplay of biological, psychological, and social factors which contribute to chronic pain (2). Specifically, children and adolescents with chronic pain endorse elevated anxiety and depressive symptoms (3, 4) increased reactivity to stress (5, 6), disrupted sleep (7), lower quality of life (8), and difficulties functioning in multiple domains (9). Unfortunately, these risk factors often interact, and it is not uncommon for youth with chronic pain to become stuck in an escalating cycle of pain, emotional distress, and disability (2, 10).

During the COVID-19 pandemic, many schools and businesses closed indefinitely in March 2020 and people were encouraged to quarantine at home to prevent further outbreak. Many children received remote education of varying quality from home, parents who were able began working from home, and most recreational and social activities outside the home (e.g., sports, clubs) were postponed or canceled. Increased emotional distress and anxiety were common across age-groups and locations during this initial period of the pandemic due to concerns about infection, social isolation, and uncertainty (11). However, some research found no change in psychosocial functioning during the pandemic (12, 13) and one study reported that a subset of participants experienced improved life satisfaction during the pandemic (14).

It is unclear how this significant disruption in daily life impacted children and adolescents with chronic pain, who may struggle to function in multiple domains due to their pain and associated distress under normal circumstances (15). However, it is possible that a reduction in functional demands due to the shutdown provided a respite to youth with chronic pain who may feel more comfortable managing their pain from home. In one study conducted in Italy, youth with chronic headache reported decreased intensity and frequency of headaches during the shutdown, which was attributed to a reduction in school-related stress and anxiety, although stress and anxiety were not assessed prior to the pandemic (16). No research could be identified in which pain and psychosocial outcomes were assessed in children and adolescents with chronic pain both prior to and during the pandemic to empirically test for change in these variables.

Anecdotally, clinicians in a multidisciplinary pediatric pain program in the northeast US observed that many of the children and adolescents receiving psychological treatment for chronic pain reported reductions in anxiety, distress, and pain intensity, and improved sleep, early in the pandemic, which could be attributed to decreased stress, more flexibility in their schedules, and reduced demands to engage in activities outside the home, especially school. Of note, in addition to the expectations of parents, teachers, and peers to function in various domains, a primary goal of multidisciplinary intervention for pediatric chronic pain is functional rehabilitation and/or maintenance (17). Continuing to function despite pain may be counterintuitive and aversive to children and adolescents with chronic pain and their parents and result in resistance and frustration with the child's treatment. Thus, the substantial reduction in expectations and demands for daily functioning due to the pandemic may have brought about a reduction in stress, at least initially. No empirical research in pediatric chronic pain could be identified assessing pain and associated psychosocial variables when functioning in multiple domains is not expected or desired. The unprecedented circumstances of prolonged shutdowns in 2020 presented a unique opportunity to evaluate potential variability in reported pain and associated psychosocial variables when stress due to functional expectations and daily schedules were reduced or eliminated.

This exploratory study aims to examine adjustment in children and adolescents with chronic pain and their families to the COVID-19 pandemic and associated shutdown. We assessed psychosocial functioning in children and adolescents with chronic pain in two naturally occurring conditions: (1) prior to the pandemic; and (2) during the initial period of shutdown related to the COVID-19 pandemic. Specifically, pain intensity and psychosocial variables (e.g., anxiety, depression, psychological stress, pain catastrophizing, sleep disturbance, and school-related quality of life) were assessed initially during a multidisciplinary evaluation in a chronic pain or headache clinic, which occurred prior to the pandemic-related shutdown. The same variables were reassessed during the initial months of COVID-19-related shutdown. Based on clinical observations, it was hypothesized that youth with chronic pain would report significant decreases in pain and improvements in psychosocial functioning, controlling for treatment effects, early in the pandemic when the need to function in multiple domains despite pain was initially minimized or eliminated.

## Methods

### Participants

Potential participants were identified by querying an IRB-approved data repository (18) which stores demographic, medical, and psychosocial data on patients with chronic pain presenting at a multidisciplinary pain treatment clinic or pediatric headache clinic in the Northeast, US. Eligibility criteria included: (1) age 8 to 18 years; (2) diagnosed with a primary chronic pain disorder (i.e., persistent or intermittent pain lasting 3 months or longer); (3) visited the clinic for initial evaluation within 10 weeks prior to the beginning of school closures, defined as January 6th to March 12th, 2020; and (4) were able to communicate in English. Exclusion criteria include: (1) Significant psychopathology (e.g., suicidality) and (2) Moderate to severe developmental delay. IRB approval was obtained to access protected health information to determine eligibility and to conduct the current study.

### Procedure

**Figure 1:**
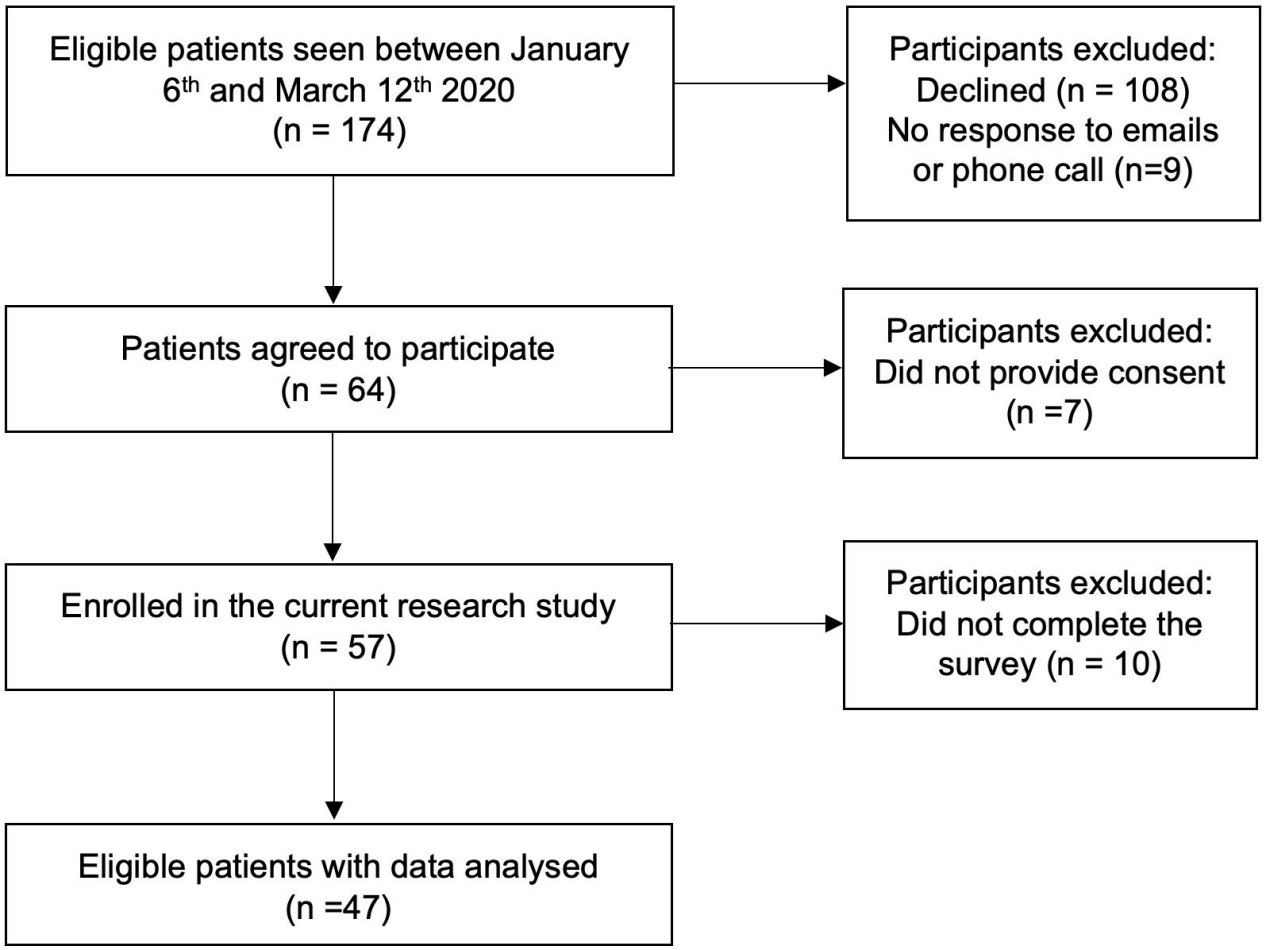
Flowchart of participants.

Eligible patients received a letter and flier by email with information about the study and how to participate. The email included a description of the study and provided the patient and parent/guardian with the option to opt out of the study if they so desired. If the patient did not opt out within 3 days, the study team proceeded with phone recruitment. If it was possible to reach the family by phone, a standard recruitment script was used to describe the study and obtain their consent, after which the study team would facilitate electronic consenting and study participation. If the family did not respond to phone calls, a standard voicemail was left describing the study and how to contact the study team. Three attempts were made to contact eligible families by phone, and if the family could not be contacted and did not return calls after three attempts, it was assumed that they were not interested in participating in the study (see Figure 1 for recruitment flowchart).

Patients who agreed to participate were provided a link allowing them to complete study measures electronically from home in accordance with social distancing requirements. Data were captured and stored in REDCap (19), a secure, HIPAA compliant web-based application. Patients were provided with a gift card in appreciation for their time after they completed the study requirements.

## Measures

Retrospective review of clinical measures administered during the patient's initial clinic evaluation prior to school closures was performed and served as baseline data (hereby referred to as “T1”). The same clinical measures were readministered, and additional COVID-19-related questionnaires were administered at follow-up during the shutdown (hereby referred to as “T2”). See Table 1 for study measures.

**Table 1:** Measures administered at T1 and T2.

**Study material**	**T1 Initial evaluation**	**T2 During COVID-19**
**Completed as part of the patient's visit to the clinic**		
PROMIS Psychological Stress	X	X
PROMIS Anxiety	X	X
PROMIS Depression	X	X
Pain Catastrophizing Scale (Child)	X	X
PedsQL—School Functioning	X	
Pain Intensity Rating	X	X
**Questionnaires added at follow-up**		
PedsQL—School Functioning		X
Treatment History Questionnaire		X
COVID-19 Impact (CEVIS)		X
Adolescent Epidemic Impact (EPII)		X

### Measures That Were Competed as a Part of the Patient's Initial Evaluation (and Were Readministered at T2)

#### Psychological Stress, Anxiety, Depressive Symptoms, and Sleep Disturbances

The Patient Reported Outcomes Measurement Information System—Short Form (PROMIS) (20, 21) is an assessment of patient reported outcomes across multiple health domains (e.g., physical symptoms, emotional distress, sleep, functioning). This study utilized the psychological stress, anxiety, depression and sleep disturbance subscales. The Short Forms each contain 8 items where youth report the frequency of an action or feeling in the past week on a scale from 1 (Never) to 5 (Almost Always). Raw scores are then converted into standardized T-scores. The PROMIS has demonstrated reliability and validity in children and adolescents ages 8–17 years (22).

#### Pain Catastrophizing

The Pain Catastrophizing Scale for Children (PCS-C) (23) is a 13-item self-report questionnaire of children's catastrophic thoughts and feelings in response to pain. Responses range from 0 (Not At All) to 4 (Extremely). Total scores range from 0 to 52 with high scores indicating greater catastrophizing. The PCS-C has been shown to be reliable and valid for children and adolescents (23, 24).

#### Pain Intensity

Patients were asked to rate their pain on a Numerical Rating Scale (NRS) that ranged from “no pain” (0) to “worst pain experienced” (10). NRS pain scores have been found to be a valid measure of pain intensity in children as young as 7 years and older (25, 26).

#### Quality of Life (School Functioning)

The Pediatric Quality of Life, Child Report (PedsQL) (27) is a 23-item self-report questionnaire assessing health-related quality of life in children and adolescents coping with acute and chronic health conditions. It includes four subscales that measure physical and psychosocial health. Only the school functioning subscale was administered in this study (5 items). Items were rated on a 5-point Likert scale from 0 (Never) to 4 (Almost Always). Scores are reverse scored and linearly transformed to a 0–100 scale with higher scores indicating better quality of life. The PedsQL has been shown to be reliable and valid for children and adolescents ages 2–18 (28).

### Additional Measures Administered During Shutdown (T2)

#### COVID-19 Impact

The COVID-19 Exposure and Family Impact Survey (CEFIS) (29) was completed by caregivers and consists of three parts assessing exposure to different aspects of the COVID-19 pandemic and the impact of the pandemic on the family. The CEFIS Exposure subscale includes 25 items assessing COVID-19-related stressors, including social aspects (e.g., inability to visit family members, canceled plans, etc.), economic aspects (e.g., lost job, lost insurance, difficulty getting food, medicine or essentials, etc.) and COVID-19-related aspects (e.g., family members exposed to or infected with COVID-19, deaths or hospitalizations of family members due to COVID-19, etc.) The Exposure score is a sum of “yes” responses ranging from a total of 0 to 25. The CEFIS Impact subscale consists of 12 items that measure the impact of COVID-19 on the family's life. Ten of the 12 items are rated on a 4-point Likert scale and 2 items are on a 10-point distress scale. The sum of the 12 items is calculated to range from 12 to 60, with a higher score indicating more negative impact or higher distress experienced due to the pandemic (see Appendix A for complete measure).

#### Adolescent Epidemic Impact

The Epidemic–Pandemic Impact Inventory (EPII)—Adolescent Adaptation (30) was developed to better understand the impact of the pandemic on various aspects of the adolescent's personal and family life (i.e., work and employment, education, homelife, social activities, economic, emotional health and well-being, physical health problems, physical distancing and quarantine, economic, infection history, and positive change). Due to overlapping content with the CEFIS, the Work and Employment, Economic, and Infection History sections were excluded from this adolescent questionnaire (see Appendix B for complete measure). The modified EPII-A comprises 84 items with “Yes,” “No” or “Not Applicable (N/A)” as possible responses. There is no standardized scoring procedure available yet; for the purpose of data analysis in this study, the sum of responses were calculated for descriptive purposes.

#### Treatment History

The Treatment History Questionnaire (THQ) is a parent-report measure assessing the range of treatments the patient has attempted between baseline and follow-up. This measure was included to allow treatment effects to be taken into account when evaluating changes in pain and psychosocial functioning. THQ includes 4 subscales that evaluate psychological, physical, medical and complementary treatment history. The parent/guardian was asked closed-ended questions that reflect whether the participant received a particular type of treatment.

### Statistical Analysis

Data analysis was conducted with IBM SPSS version 27 (31) and R. All data were screened for central tendency, variability, skewness, and kurtosis. Our a-priori power analysis conducted using G^*^Power3 (32) to test the difference between two dependent means (matched pairs) using a two-tailed test, a large effect size (*d* = 0.80), and an alpha of 0.05 indicated that a total sample of 15 participants was required to achieve a power of 0.80. Patterns of missing data and imputation was performed using the Multivariate imputation by chained equations (MICE) package in R (33). Little's test (34) was used to test whether data was missing completely at random. Sensitivity analyses were performed to compare the results of analyses with missing data with those of imputed datasets. We generated 5 imputed datasets and used 50 iterations for each imputed dataset and predictive mean matching was used to impute missing values.

Paired *t*-tests were run to compare the variables of interest at T1 and T2. To account for multiple tests, we used the Bonferroni correction and considered a *p*-value <0.05/10 = 0.005 as significant. To test whether the significant differences could be due to having received treatment in the last month, two separate repeated measures ANOVAs were run with time as the within factor and having received either physical therapy or psychotherapy as a between factor. Further, to test whether the difference in score can be attributed to potential positive effects of the COVID lockdown (based on the EPII-positive scale), a repeated measures ANCOVA was conducted with the two timepoints as the within factor and EPII as a covariate.

Change scores were computed by subtracting T1 scores from T2 scores and Pearson correlations were used to examine the relationship between the change scores, the EPII and CEFIS scales, and treatment history.

## Results

### Characteristics of Overall Sample

A flowchart of participants is presented in Figure 1. While the final sample size was adequate for the proposed analyses based on power analysis, it was smaller than we had anticipated due to difficulties with study recruitment and participation. Specifically, all research activities had to be completed remotely due to the pandemic. Remote recruitment required significantly more research personnel support and time to get families on the phone and lengthy discussion on informed consent, as well as providing assistance on technological challenges as all study procedures were completed electronically. Many families declined participation expressing screen fatigue as their children transitioned to online schooling; inability to commit to study responsibilities while balancing working from home and childcare; and the overwhelming amount of online surveys that are now required for most activities during the pandemic. Thus, it took an inordinate amount of time and effort to obtain the participants in this study.

The descriptive data are provided in Table 2. For the outcomes of interest, the amount of missing data ranged between 0 and 31.9% per variable, with a total of 22.3% missing data. The Little's test (34) was not significant, indicating that the missing data seem to be missing completely at random. Results of our sensitivity analyses indicated no significant differences between the dataset with missing values and the imputed dataset. We therefore chose to report on the non-imputed dataset.

**Table 2:** Demographic information and pain presentation in participants.

	***n***	***M* (SD)**
Age (y)	47	15.01 (2.24)
**Sex (%)**		
Female	41	87.2%
Male	6	12.8%
**Race (%)**		
White	45	95.7%
Black	1	2.1%
Latino	1	2.1%
**Parent marital status**		
Single	3	6.4%
Married	31	66%
Separated	2	4.3%
Divorced	10	21.3%
**Pain location**		
Headache	12	25.4%
Lower extremity	9	19%
Upper extremity	4	8.4%
Abdomen	2	4.3%
Chest	2	4.2%
Face/neck	4	8.5%
Pelvic	4	8.4%
Whole body	4	8.5%

Means and SDs of the study variables specific to COVID-19 are presented in Table 3. On average, participant families had a low level of exposure to COVID-19 (CEFIS Exposure *M* = 7.85, SD = 3.21) and a moderate impact due to COVID-19 (CEFIS Impact *M* = 34.92, SD = 7.38).

**Table 3:** Impact of COVID-19 on participants' personal and family life.

**COVID-19 impact questionnaires**	***M* (SD)**
**EPII**	
School (range 1–9)	5.15 (1.86)
Home (range 1–10)	2.70 (1.69)
Social (range 1–16)	6.12 (1.87)
Emotional (range 1–10)	3.51 (1.60)
Physical (range 1–12)	4.56 (2.32)
Quarantine (range 1-7)	1.73 (1.40)
Sum negative impact^*^	23.78 (6.50)
Sum positive impact^**^	10.41 (3.42)
**CEFIS**	
Exposure^***^	7.85 (3.21)
Impact^****^	34.92 (7.38)

*EPII, The Epidemic-Pandemic Impact Inventory Adolescent Adaption; CEFIS, COVID-19 Exposure and Family Impact Survey*.

* *Scores vary from 0 to 64. Higher scores higher NEGATIVE impact*.

** *May range from 0 to 20. Higher scores higher POSITIVE impact*.

*** *May range from 0 to 25*.

**** *May range from 12 to 60. Higher scores denote more negative impact*.

Overall, the adolescents reported being only modestly impacted by the COVID-19 pandemic, both negatively and positively, with the most impact reported in their social life (see Appendices A, B for frequencies of EPII and CEFIS items, respectively). In terms of school, 98% of participants attended traditional schools outside the home. During the shutdown, most adolescents reported being unable to attend school or that their school closed (85.1%), consistent with statewide mandates to shut down schools to prevent further infection. Additionally, youth endorsed having a hard time participating in virtual or distance learning from home (85.1%) and keeping up with schoolwork (85.1%). Regarding the effect of the pandemic in their family life, 85.1% of participants reported increased conflicts with parents/guardians, 76.6% reported increased conflict with siblings or other family members, and 87.2% reported less privacy and alone time. Regarding general health, adolescents reported decreased sleep quality (87.2%), using more alcohol, tobacco, vaping or other substances (61.7%), being unable to attend therapy or mental health treatment (76.6%), getting less medical care than usual (85.1%), less physical activities (85.1%), and eating more unhealthy foods (87.2%). Parents indicated that some aspects of life improved during the pandemic, while other aspects worsened (see Appendix C). For example, 60% of parents reported that family members got along better during the initial shutdown. Parents generally reported that the pandemic had a moderate impact on themselves and their children (see Table 4).

**Table 4:** CEFIS-impact rating from 1 to 10.

	*M* (SD)^*^	**Low distress *n* (%)**	**Moderate distress *n* (%)**	**High distress *n* (%)**
Overall, how much distress have you experienced related to COVID-19?	6.23 (2.08)	2 (5.0)	19 (47.5)	19 (47.5)
In general, across all your children, how much distress have your children experienced related to COVID-19?	6.35 (2.25)	4 (10.0)	15 (37.5)	21 (52.5)

* *1 indicating no distress; 10 indicating extreme distress*.

*1–3 low distress; 4–6 moderate distress; 7 and above indicating high level of distress.

Table 5 displays the means and results of the paired *t*-tests of the clinical variables of interest. On average, with exception of pain catastrophizing which was elevated at baseline, participants endorsed psychosocial symptoms in the normal to mildly elevated range at both baseline and follow-up assessments. After applying a bonferroni correction, a large and significant decrease was found in both pain intensity and pain catastrophizing between T1 and T2. Regarding the separate components of pain catastrophizing, both rumination and helplessness were significantly lower at T2 compared with T1. Magnification, the third component of pain catastrophizing, was not significantly different between T1 and T2. There were no other significant differences in the psychosocial variables between T1 and T2.

**Table 5:** Pre-COVID-19 (T1) vs. post-COVID-19 (T2) pandemic: paired sample *t*-test.

		**T1**	**T2**		
	***n***	***M* (SD)**	***M* (SD)**	**Effect size**	***p*-value**
Pain (NRS)	46	7.39 (2.13)	4.22 (2.79)	1.26	** <0.001**
PCS Total	25	26.52 (12.96)	16.56 (11.35)	0.81	**0.001**
PCS Magnification	25	3.84 (2.82)	2.80 (2.43)	0.39	0.08
PCS Rumination	25	10.04 (4.87)	6.08 (4.42)	0.85	**0.001**
PCS Helplessness	25	12.64 (6.95)	7.68 (5.83)	0.77	**0.001**
PedsQL^*^	25	46.70 (22.20)	54.60 (27)	0.32	0.20
PROMIS Psychological Stress	24	59.67 (8.53)	57.53 (5.46)	0.29	0.23
PROMIS Anxiety	25	49.94 (14.30)	50.19 (7.00)	0.02	0.93
PROMIS Depression	25	48.53 (18.09)	51.88 (8.42)	0.23	0.40
PROMIS Sleep	24	59.12 (9.85)	56.22 (9.84)	0.30	0.14

*NRS, 0–10 Numeric Rating Scale; PCS, Pain Catastrophizing Scale; PedsQL, Pediatric Quality of Life; PROMIS, Patient Reported Outcomes Measurement Information System. Data were analyzed using paired t-tests using Bonferroni correction to adjust multiple comparisons. A p-value <0.005 was considered significant (bold). Effect Sizes correspond to Cohen's D*.

* *PedsQL school functioning subscale*.

No significant interaction was found between EPII and any outcome. No significant interaction was found between having received treatment and any outcome, nor was there any significant between-subjects effect. All outcomes remained statically significant when controlling for treatment except PCS-Helpless, which was reduced to a trend level significance (*p* = 0.066) when controlling for having received physical therapy in the past 2 months.

Table 6 presents the pattern of correlations among the change scores for the variables of interest. Changes in pain catastrophizing between T1 and T2 were positively correlated with change in psychological stress (*r* = 0.57, *p* = 0.004) and anxiety (*r* = 0.54, *p* = 0.005) and negatively correlated with changes in quality of life (*r* = −0.45, *p* = 0.024) and the EPII positive scale (*r* = −0.40, *p* = 0.049). Change in psychological stress was positively associated with change in anxiety (*r* = 0.59, *p* = 0.002) and depression (*r* = 0.49, *p* = 0.014).

**Table 6:** Correlations between selected variables.

**Variables**		**1**	**2**	**3**	**4**	**5**	**6**	**7**	**8**	**9**
1	NRS	1.00								
2	PCS	−0.08	1.00							
3	PedsQL	−0.05	−0.45^*^	1.00						
4	PROMIS Psych	0.02	0.57^**^	−0.24	1.00					
5	PROMIS Anxiety	0.04	0.54^**^	−0.12	0.59^**^	1.00				
6	PROMIS Depression	0.28	0.15	−0.04	0.49^*^	0.29	1.00			
7	PROMIS Sleep	0.15	0.17	−0.27	0.23	0.11	0.26	1.00		
8	EPII Positive	0.16	−0.40^*^	0.25	−0.04	0.07	−0.22	−0.39	1.00	
9	EPII Negative	0.06	0.39	−0.02	0.26	0.25	−0.02	0.22	−0.20	1.00

*Significant correlations are marked in bold*.

** *p <0.01 (two-tailed)*;

* *p <0.05 (two-tailed)*.

## Discussion

Children and adolescents with chronic pain may experience difficulties functioning in multiple domains, and stress due to functional difficulties may result in increased pain and anxiety, disrupted sleep, and ongoing functional disability (35). The COVID-19 pandemic in 2020 resulted in a unique circumstance during which schools, business, and activities across the US were closed for several months, and people were advised to remain at home, to prevent the spread of the virus. This unprecedented situation allowed for a comparison of pain intensity, pain-related outcomes, and psychosocial adjustment in youth with chronic pain under two naturally occurring conditions: when functioning outside the home was the norm prior to the pandemic and during prolonged home quarantine and shutdown due to the pandemic. Based on our clinical observations, we hypothesized that children and adolescents with chronic pain would endorse decreased pain, pain-related impairment, and psychosocial distress, and improved functioning early in the pandemic when the expectation that they continue to function in school and other activities despite pain was reduced or eliminated during the initial period of pandemic-related shutdown.

Children and adolescents with chronic pain and their parents endorsed both positive and negative aspects of the pandemic. Positive aspects included improvement in relationships with family and friends, greater flexibility in school, less peer pressure, and more time outside. As expected, many reported that they were not able to attend school in person. Negative aspects included difficulties participating in remote school, trouble keeping up with school, increased family stress, reduced time with peers, unhealthy sleep and eating habits, and reduced access to treatment. Thus, they continued to experience school and family stress in the context of less healthy habits and a disruption in their care. Overall, families described a low level of exposure to COVID-19 related stressors and moderate impact of the pandemic. In general, families in this sample experienced disruption to their daily lives due to school and office closures and home quarantine but did not experience severe pandemic-related stress such as deaths in the family, lack of income, or loss of housing.

As predicted, reported pain intensity decreased significantly during the first few months of the shutdown, as compared to prior to the pandemic several weeks before. It is certainly possible that the decrease in pain intensity occurred as a result of education about chronic pain and treatment received during the initial clinic visit prior to the pandemic, although the amount of decrease in pain intensity is not typical for the clinic population. It is also possible that the physical, cognitive, and social demands of attending school in-person and participating in extracurricular and social activities outside the home may contribute to pain intensity in children and adolescents with chronic pain. The school environment, in particular, may be challenging for youth with chronic pain for several reasons, including the set schedule, need to remain alert and engaged, and physical challenges of sitting still for a full class period and navigating from class to class in crowded hallways. It may be that the added flexibility in schedule and pain management options while at home allowed youth to manage their pain better, resulting in reduced pain. Peer relationships are an additional source of stress in school. Youth with chronic pain experience greater loneliness and depressive symptoms in the context of their friendships (36) and poorer social functioning is linked with increased pain and school impairment in youth with chronic pain (37). Thus, it may be that withdrawal from academic and social stress in school was associated with decreased pain directly, even when additional stressors related to the pandemic (e.g., increased family conflict, social isolation) were present. There may also be a behavioral component, as elevated pain intensity may be inadvertently reinforced with withdrawal from the potentially stressful academic and social environments. When the reinforcement (i.e., remaining home) becomes the norm rather than the exception, pain may gradually decrease.

Furthermore, pain catastrophizing, including rumination and helplessness, was also significantly decreased during the initial shutdown, as compared to several weeks earlier prior to the pandemic. Thus, children and adolescents with chronic pain reported less excessive, negative thinking about their pain during the first few months of the pandemic when they were not required or expected to engage in activities outside the home. Surprisingly, change in pain catastrophizing was not associated with change in pain intensity, suggesting these variables operate independently. It may be that managing chronic pain outside the home, particularly in the school setting where academic and social expectations are high and opportunities for individual flexibility are limited, is especially challenging, leading to increased rumination and helplessness. When children and adolescents with chronic pain have the ability to choose how to structure their environment and schedule in a way that's most beneficial to them, including flexibility in their activities and the ability to employ pain coping strategies as needed, they may be less likely to catastrophize about pain.

A decrease in psychological stress was associated with a decrease in anxiety and pain catastrophizing during the initial period of pandemic-related shutdown. That is, if there is a general reduction in stress, children and adolescents with chronic pain are less anxious and engage in less catastrophic thinking about their pain. Furthermore, there appears to be an improvement in general quality of life that is associated with decreased general stress. The most significant change for children and adolescents during this time period was the sudden closure of schools, which likely resulted in a decrease in both academic and social stress, initially. Participants continued to experience stress related to the pandemic and prolonged quarantine with their families; however, a reduction in stress overall, most likely attributed to reduced demands for functioning outside the home, was linked to improvement in emotional adjustment and quality of life. As the pandemic persists and the shutdown is extended, children and adolescents have generally reported increased emotional distress and poorer quality of life due to social isolation and activity limitations (38). A future goal of this study is to reassess participant's psychosocial adjustment after a year of school closures.

It is notable that a decrease in pain was not associated with participation in physical therapy or psychotherapy. Unfortunately, many outpatient CBT and PT practices were also forced to close during the pandemic, resulting in a disruption in treatment for many pediatric chronic pain patients. Indeed, three quarters of participants reported that they had less access to mental health care and 85% endorsed decreased access to general medical care. The lack of availability of health care, particularly mental health care, during the pandemic may contribute to poorer long-term adjustment in youth. The clinical implications of these findings are complex. The initial reaction may be to remove children and adolescents with chronic pain from traditional school settings and activities outside the home to reduce functional demands and pain. While this might be appropriate for some individuals, it is not the most beneficial plan for all youth with chronic pain. The domains in which children and adolescents with chronic pain are expected to function, particularly school, are a microcosm that prepares them for life in the larger world and avoiding activities outside the home will not serve the developmental needs of most children in the long run. Rather, findings reinforce the importance of teaching effective pain coping strategies combined with self-advocacy skills so that youth with chronic pain have the skills and resources to manage their pain in whichever environment in which they are expected to function. Additionally, results of this study suggest that restructuring activities, especially the school day, to make it more supportive of all students, including those with chronic pain, would likely be beneficial. For example, there have been efforts to shift school start times later to coincide with adolescents' natural circadian rhythms and allow for improved sleep health. Additional flexibility in scheduling and environmental factors (e.g., comfortable chairs, access to remote learning technology) may also be helpful (39). Additionally, there are ways that teachers and coaches can interact with students with chronic pain that validate their pain while also supporting and encouraging optimal functioning (39), and these strategies should be promoted.

There are several significant limitations to this study which are worth noting. First, findings are based on a relatively small sample of treatment-seeking children and adolescents with chronic pain in the Northeast, who were primarily female and Caucasian, and results may not generalize to diverse youth with chronic pain in other locations. Second, there may be a selection bias, as those who agreed to participate in this study may have had more positive experiences during school closures than those who declined to participate. Third, we did not directly assess stress related to functioning in various domains outside the home, especially school, so we cannot be certain that changes in pain intensity and pain catastrophizing were due to reduced functional demands. While reduced functional expectations outside the home was a major change from the first time point to the second, there were other changes as well-related to the COVID-19 pandemic which may have contributed to these findings, although notably, the sample as a whole reported low levels of some of the more severe COVID-19 effects (e.g., hospitalization or deaths in the family). Lastly, there may be additional factors which contributed to the clinical changes, including education and feedback from the initial pain clinic evaluation and improvement due to the natural course of the child's pain.

## Conclusion

In summary, the results of this study highlight both the positive and negative aspects of being required to remain at home due to a global pandemic for children and adolescents with chronic pain and their families. The fact that pain intensity and pain catastrophizing decreased significantly during the initial shutdown suggests that stress related to functional demands outside the home contributes to pain and pain-related outcomes. While it is not advisable to withdraw children and adolescents from school and other activities due to their chronic pain, results do suggest that some environmental changes may be warranted to allow all children and adolescents, including those with chronic pain, to optimize their academic, emotional, and social development and growth. Furthermore, a curriculum for all students focused on stress management and socioemotional development would help youth manage the challenges inherent in growing up more successfully. While these findings are based on a small sample of youth with chronic pain, it may be that greater flexibility and individuality in activities outside the home, especially school, would benefit children and adolescents in general. More research with diverse samples is needed to determine if these findings are generalizable beyond children and adolescents with chronic pain.

## Data Availability Statement

The raw data supporting the conclusions of this article will be made available by the authors, without undue reservation.

## Ethics Statement

The studies involving human participants were reviewed and approved by Boston Children's Hospital IRB. Written informed consent to participate in this study was provided by the participants' legal guardian/next of kin.

## Author Contributions

KK contributed the initial study hypothesis, study design, and extensive manuscript writing and revising. JK contributed hypothesis generation, study design, data analysis, and manuscript writing. CC and CK contributed study design, data collection and management, and manuscript writing. JC contributed data collection and analysis and manuscript revision. CBB, DL, and SN contributed guidance on study design and manuscript review. All authors contributed to the article and approved the submitted version.

## Funding

This study was supported by the Sara Page Mayo Endowment for Pediatric Pain Research Education and Treatment.

## Conflict of Interest

The authors declare that the research was conducted in the absence of any commercial or financial relationships that could be construed as a potential conflict of interest.

## Publisher's Note

All claims expressed in this article are solely those of the authors and do not necessarily represent those of their affiliated organizations, or those of the publisher, the editors and the reviewers. Any product that may be evaluated in this article, or claim that may be made by its manufacturer, is not guaranteed or endorsed by the publisher.

## References

[1] Huguet A Miró J. The severity of chronic pediatric pain: an epidemiological study. J Pain. (2008) 9:226–36. 10.1016/j.jpain.2007.10.015 18088558

[2] Vetter TR McGwin G Jr Bridgewater CL Madan-Swain A Ascherman LI. Validation and clinical application of a biopsychosocial model of pain intensity and functional disability in patients with a pediatric chronic pain condition referred to a subspecialty clinic. Pain Res Treat. (2013) 2013:143292. 10.1155/2013/143292 24251035 PMC3819919

[3] Benore E D'Auria A Banez GA Worley S Tang A. The influence of anxiety reduction on clinical response to pediatric chronic pain rehabilitation. Clin J Pain. (2015) 31:375–83. 10.1097/AJP.0000000000000127 24977393

[4] Kashikar-Zuck S Goldschneider KR Powers SW Vaught MH Hershey AD. Depression and functional disability in chronic pediatric pain. Clin J Pain. (2001) 17:341–9. 10.1097/00002508-200112000-00009 11783815

[5] Nelson S Burns M Logan D. The clinical utility of a brief psychological stress measure (patient-reported outcomes measurement information system) in youth with chronic pain. Pain Med. (2021) 22:91–9. 10.1093/pm/pnaa263 32914177

[6] Varni JW Stucky BD Thissen D Dewitt EM Irwin DE Lai JS. PROMIS Pediatric Pain Interference Scale: an item response theory analysis of the pediatric pain item bank. J Pain. (2010) 11:1109–19. 10.1016/j.jpain.2010.02.005 20627819 PMC3129595

[7] Valrie CR Bromberg MH Palermo T Schanberg LE. A systematic review of sleep in pediatric pain populations. J Dev Behav Pediatr. (2013) 34:120–8. 10.1097/DBP.0b013e31827d5848 23369958 PMC3562475

[8] Gold JI Yetwin AK Mahrer NE Carson MC Griffin AT Palmer SN. Pediatric chronic pain and health-related quality of life. J Pediatr Nurs. (2009) 24:141–50. 10.1016/j.pedn.2008.07.003 19268235

[9] Liossi C Howard RF. Pediatric chronic pain: biopsychosocial assessment and formulation. Pediatrics. (2016) 138:e20160331. 10.1542/peds.2016-0331 27940762

[10] Wendland M Jackson Y Stokes LD. Functional disability in paediatric patients with recurrent abdominal pain. Child Care Health Dev. (2010) 36:516–23. 10.1111/j.1365-2214.2010.01087.x 20412144

[11] Acheson R. Research digest: the impact of the Covid-19 pandemic on child, adolescent, young adult, and family mental health. J Child Psychother. (2020) 46:429–40. 10.1080/0075417X.2021.1912810

[12] Xiang Y-T Yang Y Li W Zhang L Qinge Z Cheung T. Timely mental health care for the 2019 novel coronavirus outbreak is urgently needed. Lancet Psychiat. (2020) 7:228–9. 10.1016/S2215-0366(20)30046-8 32032543 PMC7128153

[13] Isumi A Doi S Yamaoka Y Takahashi K Fujiwara T. Do suicide rates in children and adolescents change during school closure in Japan? The acute effect of the first wave of COVID-19 pandemic on child and adolescent mental health. Child Abuse Negl. (2020) 110:104680. 10.1016/j.chiabu.2020.104680 32847679 PMC7443207

[14] Tang S Xiang M Cheung T Xiang Y-T. Mental health and its correlates among children and adolescents during COVID-19 school closure: the importance of parent-child discussion. J Affect Disord. (2021) 279:353–60. 10.1016/j.jad.2020.10.016 33099049 PMC7550131

[15] Miller MM Meints SM Hirsh AT. Catastrophizing, pain, and functional outcomes for children with chronic pain: a meta-analytic review. Pain. (2018) 159:2442–60. 10.1097/j.pain.0000000000001342 30015710 PMC6237640

[16] Papetti L Loro PAD Tarantino S Grazzi L Guidetti V Parisi P. I stay at home with headache. A survey to investigate how the lockdown for COVID-19 impacted on headache in Italian children. Cephalalgia. (2020) 40:1459–73. 10.1177/0333102420965139 33146039 PMC7684684

[17] Liossi C Johnstone L Lilley S Caes L Williams G Schoth DE. Effectiveness of interdisciplinary interventions in paediatric chronic pain management: a systematic review and subset meta-analysis. Br J Anaesth. (2019) 123:e359–71. 10.1016/j.bja.2019.01.024 30916012 PMC6676017

[18] Donado C Lobo K Berde C Bourgeois F. Developing a pediatric pain data repository. JAMIA Open. (2019) 3:31–6. 10.1093/jamiaopen/ooz062 32607485 PMC7309240

[19] Harris P Taylor R Thielke R Payne J Gonzalez N Conde J. Research Electronic Data Capture (REDCap) - a metadata-driven methodology and workflow process for providing translational research informatics support. J Biomed Informat. (2008) 42:377–81. 10.1016/j.jbi.2008.08.010 18929686 PMC2700030

[20] Cella D Riley W Stone A Rothrock N Reeve B Yount S. The patient-reported outcomes measurement information system (PROMIS) developed and tested its first wave of adult self-reported health outcome item banks: 2005–2008. J Clin Epidemiol. (2010) 63:1179–94. 10.1016/j.jclinepi.2010.04.011 20685078 PMC2965562

[21] Schalet BD Pilkonis PA Yu L Dodds N Johnston KL Yount S. Clinical validity of PROMIS Depression, Anxiety, and Anger across diverse clinical samples. J Clin Epidemiol. (2016) 73:119–27. 10.1016/j.jclinepi.2015.08.036 26931289 PMC4928679

[22] Hinds PS Nuss SL Ruccione KS Withycombe JS Jacobs S DeLuca H. PROMIS pediatric measures in pediatric oncology: valid and clinically feasible indicators of patient-reported outcomes. Pediatr Blood Cancer. (2013) 60:402–8. 10.1002/pbc.24233 22829446

[23] Crombez G Bijttebier P Eccleston C Mascagni T Mertens G Goubert L. The child version of the pain catastrophizing scale (PCS-C): a preliminary validation. Pain. (2003) 104:639–46. 10.1016/S0304-3959(03)00121-0 12927636

[24] Vervoort T Eccleston C Goubert L Buysse A Crombez G. Children's catastrophic thinking about their pain predicts pain and disability 6 months later. Eur J Pain. (2010) 14:90–6. 10.1016/j.ejpain.2009.03.001 19359203

[25] von Baeyer CL. Children's self-reports of pain intensity: scale selection, limitations and interpretation. Pain Res Manag. (2006) 11:157–62. 10.1155/2006/197616 16960632 PMC2539005

[26] Voepel-Lewis T Burke CN Jeffreys N Malviya S Tait AR. Do 0-10 numeric rating scores translate into clinically meaningful pain measures for children? Anesth Analg. (2011) 112:415–21. 10.1213/ANE.0b013e318203f495 21127278

[27] Varni JW Seid M Rode CA. The PedsQL: measurement model for the pediatric quality of life inventory. Med Care. (1999) 37:126–39. 10.1097/00005650-199902000-00003 10024117

[28] Varni JW Seid M Kurtin PS. PedsQL 4.0: reliability and validity of the Pediatric Quality of Life Inventory version 4.0 generic core scales in healthy and patient populations. Med Care. (2001) 39:800–12. 10.1097/00005650-200108000-00006 11468499

[29] Kazak AE Alderfer M Enlow PT Lewis AM Vega G Barakat L. COVID-19 exposure and family impact scales: factor structure and initial psychometrics. J Pediatr Psychol. (2021) 46:504–13. 10.1093/jpepsy/jsab026 33749794 PMC8083683

[30] Morris AS Ratliff EL Grasso DJ Briggs-Gowan MJ Ford JD Carter AS. The Epidemic – Pandemic Impacts Inventory Adolescent Adaptation (EPII-A). Farmington, CT: University of Connecticut School of Medicine (2020).

[31] Corp I. IBM SPSS Statistics for Windows. 27.0 ed. Armonk, NY: IBM Corp (2020).

[32] Faul F Erdfelder E Lang AG Buchner A. G^*^Power 3: a flexible statistical power analysis program for the social, behavioral, and biomedical sciences. Behav Res Methods. (2007) 39:175–91. 10.3758/BF03193146 17695343

[33] Buuren Sv. Flexible Imputation of Missing Data. 2nd ed. Boca Raton, FL: Chapman and Hall/CRC Press (2018).

[34] Little RJA. A test of missing completely at random for multivariate data with missing values. J Am Stat. (1988) 83:1198–202. 10.1080/01621459.1988.10478722

[35] Solé E Galán S de la Vega R Castarlenas E Sánchez-Rodríguez E Jensen MP. Psychometric properties of the Functional Disability Inventory for assessing Pain-related disability in children from the community. Disabil Rehabil. (2019) 41:2451–8. 10.1080/09638288.2018.1467969 29697002

[36] Forgeron PA Chambers CT Cohen J Dick BD Finley GA Lamontagne C. Dyadic differences in friendships of adolescents with chronic pain compared with pain-free peers. Pain. (2018) 159:1103–11. 10.1097/j.pain.0000000000001191 29474206

[37] Simons LE Logan DE Chastain L Stein M. The relation of social functioning to school impairment among adolescents with chronic pain. Clin J Pain. (2010) 26:16–22. 10.1097/AJP.0b013e3181b511c2 20026948

[38] Imran N Aamer I Sharif MI Bodla ZH Naveed S. Psychological burden of quarantine in children and adolescents: a rapid systematic review and proposed solutions. Pak J Med Sci. (2020) 36:1106–16. 10.12669/pjms.36.5.3088 32704298 PMC7372688

[39] Vervoort T Logan DE Goubert L De Clercq B Hublet A. Severity of pediatric pain in relation to school-related functioning and teacher support: an epidemiological study among school-aged children and adolescents. Pain. (2014) 155:1118–27. 10.1016/j.pain.2014.02.021 24631587

