# Supplementary material for: Initial Adjustment to the COVID-19 Pandemic and the Associated Shutdown in Children and Adolescents With Chronic Pain and Their Families
Source: Front Pain Res (Lausanne). 2021 Sep 30;2:713430. doi: 10.3389/fpain.2021.713430 (PMC8915775; doi:10.3389/fpain.2021.713430)
Supplement: Supplementary file 1 [file Data_Sheet_1.docx]

| **APPENDIX A.** Participant Report of EPII Items |  |
| --- | --- |
|  | n (%) |
| School |  |
| School closed or was unable to go to school | 39 (97.5) |
| Hard time participating in virtual or distance learning from home | 18 (45) |
| Hard time keeping up with schoolwork | 19 (47.5) |
| Unable to attend important school events | 28 (73.7) |
| Unable to attend after school activities | 28 (83.8) |
| Unable to participate in school athletics | 28 (90.3) |
| Unable to participate in community or non-school related clubs and organizations | 30 (78.9) |
| Unable to complete important life steps | 16 48.5 |
| Returned home from college, boarding school study abroad, or other away-from home living situation | 2 (12.5) |
| Home |  |
| Difficulty taking care of siblings or other children in the home | 6 (23.2) |
| Had to spend time teaching or helping a sibling do schoolwork | 9 (29) |
| Changes in responsibilities or chores at home | 25 (64.1) |
| More conflict with parent(s) or other adults who look after me | 20 (50) |
| More conflict with siblings or other family members | 22 (61.1) |
| Limited privacy or alone time | 22 (53.7) |
| Family or friends had to move into my home | 3 (8.8) |
| Had to spend a lot more time taking care of an adult family member | 1 (2.8) |
| Had to move or relocate | 3 (8.3) |
| Became homeless | 0 |
| Social Activities |  |
| Separated from family or family member | 10 (25) |
| Separated from friend(s) | 10 (97.6) |
| Separated from a girlfriend/boyfriend or romantic partner | 4 (15.4) |
| Had more arguments or conflicts with friends | 6 (15.4) |
| Had more arguments or conflict with a girlfriend/boyfriend or romantic partner | 1 (4.5) |
| Increased bullying or harassment on phone texts or social media | 4 (10.8) |
| Broke-up with a girlfriend/boyfriend or romantic partner | 3 (12) |
| Did not have the ability or resources to talk to family, friends, or a girlfriend/boyfriend or romantic partner while separated | 4 (10) |
| Unable to visit a loved one in a care facility | 9 (34.6) |
| Family celebrations cancelled or restricted | 36 (92.3) |
| Planned travel or vacations cancelled | 31 (79.5) |
| Religious or spiritual activities cancelled or restricted | 25 (71.4) |
| Unable to be with a close family member in critical condition | 5 (20) |
| Unable to attending person funeral or religious services for a family member or friend who died | 6 (25) |
| Unable to participate in social clubs, sports teams, or usual volunteer activities | 36 (92.3) |
| Unable to do enjoyable activities or hobbies | 31 (75.6) |
| Emotional Health and Well-Being |  |
| Got into trouble more often | 6 (14.6) |
| Had increased sleep difficulties, poor sleep quality, or nightmares | (53.7) |
| Had increased mental health problems or symptoms | 30 (73.2) |
| Used more alcohol, tobacco, vaping, or other substances | 0 |
| Unable to access mental health treatment or therapy | 9 (25) |
| Not satisfied with changes in mental health treatment or therapy | 7 (20) |
| Spent more time on screens and devices | 40 (97.6) |
| Parent had increased mental health problems or symptoms | 20 (52.6) |
| Parent increased use of alcohol or substances | 6 (17.6) |
| Parent unable to access mental health treatment or therapy | 4 (12.1) |
| Physical Health Problems |  |
| Increased health problems not related to this disease | 14 (34.1) |
| Less physical activity or exercise | 31 (77.5) |
| Overate or ate more unhealthy foods | 18 (43.9) |
| Spent more time sitting down or being sedentary | 32 (78) |
| Important medical procedure cancelled | 12 (32.4) |
| Unable to access medical care for a serious condition | 9 (28.1) |
| Got less medical care than usual | 31 (77.5) |
| Elderly or disabled family member not in the home unable to get the help they need | 6 (23.1) |
| Parent(s) had increased health problems not related to this disease | 7 (19.4) |
| Parent(s) important medical procedures were cancelled | 7 (21.2) |
| Parent(s) unable to access medical care for a serious condition | 5 (16.1) |
| Parent(s) got less medical care than usual | 15 (39.5) |
| Physical Distance and Quarantine |  |
| Isolated or quarantined due to possible exposure to this disease | 9 (22) |
| Isolated or quarantined due to symptoms of this disease | 6 (15) |
| Isolated due to existing health conditions that increase risk of infection or disease | 15 (37.5) |
| Had limited physical closeness with a parent or loved one due to concerns of infection | 17 (42.5) |
| A close family member not in the home was quarantined | 10 (25.6) |
| A family member was unable to return home due to quarantine or travel restrictions | 3 (7.9) |
| Entire household was quarantined for a week or longer | 11 (27.5) |
| Positive Change |  |
| More quality time with family, friends, or romantic partner in person or from a distance | 35 (85.4) |
| More quality time with parent(s) or other adults who look after me at home | 35 (85.4) |
| More quality time with siblings and other family members | 32 (91.4) |
| Improved relationships with family, friends, or a romantic partner | 23 (56.1) |
| New connections made with supportive people | 20 (52.6) |
| Spent more time playing and caring for pet(s) | 31 (83.8) |
| Increase in exercise or physical activity | 12 (30) |
| More time in nature or being outdoors | 26 (63.4) |
| More time doing enjoyable activities | 28 (68.3) |
| Developed new hobbies or activities | 26 (63.4) |
| More appreciative of things usually taken for granted | 31 (75.6) |
| Paid more attention to personal health | 31 (77.5) |
| Paid more attention to preventing physical injuries | 18 (46.2) |
| Ate healthier foods | 21 (51.2) |
| Less use of alcohol, tobacco, vaping, or other substances | 4 (66.7) |
| Spent less time on screens or devices outside of work hours | 6 (14.6) |
| Volunteered time to help people in need | 8 (21.6) |
| Donated time or goods to a cause related to this disease | 11 (27.5) |
| Found greater meaning in work or school | 13 (32.5) |
| More efficient or productive in work or school | 16 (40) |
