# Supplementary material for: Initial Adjustment to the COVID-19 Pandemic and the Associated Shutdown in Children and Adolescents With Chronic Pain and Their Families
Source: Front Pain Res (Lausanne). 2021 Sep 30;2:713430. doi: 10.3389/fpain.2021.713430 (PMC8915775; doi:10.3389/fpain.2021.713430)
Supplement: Supplementary file 2 [file Data_Sheet_2.docx]

| **APPENDIX B.** Participant Report of CEFIS-Exposure Items | |
| --- | --- |
|  | n (%) |
| "Stay at home" order | 34 (85) |
| Schools/child care centers were closed | 39 (97.5) |
| Children's education was disrupted | 38 (95) |
| Unable to visit or care for a family member | 27 (67.6) |
| Our family lived separately for health, safety or job demands | 9 (22.5) |
| Someone moved into (or back into) their home | 8 (20) |
| Move out of our home | 1 (2.5) |
| Someone in the family kept working outside the home | 18 (45) |
| Someone in the family is a healthcare provider/first responder providing direct care | 8 (20) |
| Difficulty getting food | 5 (12.5) |
| Difficulty getting medicine | 2 (5) |
| Difficulty getting health care when we needed it | 11 (27.5) |
| Difficulty getting other essentials | 9 (22.5) |
| Self-quarantined due to travel or possible exposure | 14 (35) |
| Decreased family income | 17 (42.5) |
| Family member had to cut back hours at work | 13 (32.5) |
| A member of the family was required to stop working | 9 (22.5) |
| A member of the family lost their job permanently | 4 (10) |
| Loss of health insurance/benefits | 3 (7.5) |
| We missed an important family event or it was cancelled | 34 (85) |
| Someone in the family was exposed to someone with COVID-19 | 3 (7.5) |
| Someone in the family had symptoms or was diagnosed with COVID-19 | 3(7.5) |
| Someone in the family was hospitalized for COVID-19 | 2 (5) |
| Someone in the family was in the Intensive Care Unit (ICU) for COVID-19 | 1 (2.5) |
| Someone in the family died from COVID-19 | 1 (5) |
