# Supplementary material for: Initial Adjustment to the COVID-19 Pandemic and the Associated Shutdown in Children and Adolescents With Chronic Pain and Their Families
Source: Front Pain Res (Lausanne). 2021 Sep 30;2:713430. doi: 10.3389/fpain.2021.713430 (PMC8915775; doi:10.3389/fpain.2021.713430)
Supplement: Supplementary file 3 [file Data_Sheet_3.docx]

| **APPENDIX C.** Participant Report of CEFIS-Impact Items | | | | |
| --- | --- | --- | --- | --- |
|  | n (%) | | | |
|  | Made it a lot better | Made it a little better | Made it a little worse | Made it a lot worse |
| **Impact** | | | | |
| Parenting | 5 (12.5) | 12 (30) | 21 (52.5) | 2 (5) |
| How family members get along with each other | 5 (12.5) | 19 (47.5) | 16 (40) | 0 (0) |
| Ability to care for your child with chronic pain | 8 (20) | 16 (40) | 10 (25) | 6 (15) |
| Ability to care for other children in your family | 6 (15) | 20 (50) | 9 (22.5) | 5 (12.5) |
| Ability to care for older adults or people with disabilities in your family | 4 (10) | 11 (27.5) | 19 (47.5) | 6 (15) |
| Your physical wellbeing - exercise | 3 (7.5) | 11 (27.5) | 18 (45) | 8 (20) |
| Your physical wellbeing - eating | 5 (12.5) | 10 (25) | 17 (42.5) | 8 (20) |
| Your physical wellbeing - sleeping | 3 (7.5) | 10 (25) | 18 (45) | 9 (22.5) |
| Your emotional wellbeing - anxiety | 2 (5) | 2 (5) | 28 (70) | 8 (20) |
| Your emotional wellbeing - mood | 1 (2.5) | 8 (20) | 23 (57.5) | 8 (20) |
